# Correlation analysis and clinical significance of Musculoskeletal Ultrasound semi-quantitative grading with bone salt metabolism, Rheumatoid factor and Erythrocyte Sedimentation rate in patients with Rheumatoid Arthritis

**DOI:** 10.12669/pjms.39.6.7144

**Published:** 2023

**Authors:** Pei-ze Song, Nan Wu, Cong Huo, Xiao-ming Wang

**Affiliations:** 1 Pei-ze Song, Dept. of Integrated Traditional Chinese and Western Medicine, Baoding NO.1 Central Hospital, Baoding 071000, Hebei, P.R. China; 2 Nan Wu, Dept. of Integrated Traditional Chinese and Western Medicine, Baoding NO.1 Central Hospital, Baoding 071000, Hebei, P.R. China; 3 Cong Huo, Dept. of Integrated Traditional Chinese and Western Medicine, Baoding NO.1 Central Hospital, Baoding 071000, Hebei, P.R. China; 4 Xiao-ming Wang, Dept. of Integrated Traditional Chinese and Western Medicine, Baoding NO.1 Central Hospital, Baoding 071000, Hebei, P.R. China

**Keywords:** Musculoskeletal ultrasound semi-quantitative grading, Bone metabolism index, Erythrocyte sedimentation rate, Rheumatoid factor, Diagnosis

## Abstract

**Objective:**

To determine the correlation and clinical significance of musculoskeletal ultrasound semi-quantitative grading with bone salt metabolism, rheumatoid factor and erythrocyte sedimentation rate (ESR) in patients with rheumatoid arthritis.

**Methods:**

This is a clinical comparative study. A total of 240 patients with rheumatoid arthritis admitted to Baoding NO.1 Central Hospital were selected according to the DAS28 score of rheumatoid arthritis, and were divided into four groups, with 60 cases in each group from May 2020 to May 2022. The differences and correlation of musculoskeletal ultrasound semi-quantitative grading, bone metabolism indicators, erythrocyte sedimentation rate and rheumatoid factor among the four groups were statistically analyzed.

**Results:**

The scores of bone erosion, synovial hyperplasia, joint effusion and intrasynovial blood flow in Group-H were significantly higher than those in Group-R, L and M, with statistically significant differences(p=0.00). The procollagen Type-1 N-terminal propeptide(P1NP), bone-specific alkaline phosphatase(BALP) and osteoprotegerin(OPG) in Group-H were significantly lower than those in Group-R, L and M, with statistically significant differences(p=0.00); The tartrate-resistant acid phosphatase(TRAC) in Group-H was significantly higher than that in Group-R, L and M, with a statistically significant difference(p=0.00). The levels of RF and ESR in Group-H were significantly higher than those in Group-R, L and M, with statistically significant differences(p=0.00).

**Conclusion:**

Musculoskeletal ultrasound semi-quantitative grading is correlated with the level of bone salt metabolism, rheumatoid factor and ESR in patients with rheumatoid arthritis. It can be combined with laboratory examination to objectively judge the severity of the course of rheumatoid arthritis.

## INTRODUCTION

Rheumatoid arthritis (RA) is a common clinical chronic inflammatory autoimmune disease that may give rise to synovitis, joint pannus, cartilage and bone destruction, and ultimately lead to joint deformity and disability.^1^ Currently, the pathogenesis of RA has not been fully clarified. It is generally believed that the occurrence and development of RA are based on immune damage and repair caused by immune-mediated susceptibility gene participation, infectious factors and autoimmune reaction. Genetic and environmental factors determine susceptibility to RA.^2^ According to studies, inflammatory factors play a vital role in promoting synovitis and pannus formation in patients with RA. In RA active stage, a large amount of interleukin, CRP, tumor necrosis factor (TNF) and other inflammatory factors permeate the synovium to promote inflammatory injury^3^, resulting in progressive damage and deformity of joints. Consequently, accurate diagnosis and effective treatment measures should be taken as early as possible to improve the physical and mental health of patients and ameliorate their quality of life. At present, laboratory examinations and corresponding imaging examinations such as joint X-ray examinations and MRI examinations are mainly used for the diagnosis of RA.

Musculoskeletal ultrasound (MSUS) is an emerging ultrasound technique in recent years which is used to diagnose diseases of the musculoskeletal system by means of high-frequency ultrasound. It can clearly display the superficial soft tissue structures such as muscles, tendons, ligaments and peripheral nerves and their lesions, such as structural abnormalities caused by inflammation, tumor, injury and deformity.^4^ As a sensitive imaging modality used by clinicians to assist in making decisions for the treatment of rheumatoid arthritis (RA)^5^, MSUS is characterized by non-invasive, painless, and operator-friendly features.^6^ In this study, the semi-quantitative grading of MSUS was combined with common diagnostic indicators of RA, such as bone salt metabolism, rheumatoid factor and ESR, to evaluate the correlation of the above indicators, confirming that MSUS has certain significance in clinical diagnosis and condition evaluation.

## METHODS

This is a clinical comparative study. A total of 240 patients with rheumatoid arthritis admitted to Baoding NO.1 Central Hospital were selected according to the DAS28 score of rheumatoid arthritis (28-joint disease activity)^7^ and were divided into four groups: remission stage group (R-group), low activity stage group (L-group), moderate activity stage group (M group) and high activity stage group (H group), with 60 cases in each group from May 2020 to May 2022 . There were 41 males and 19 females in Group-R, aged 23-60 years, with an average of 53.83±7.08 years; There were 43 males and 17 females in L-group, aged 19-62 years, with an average of 52.76±7.35 years. Group-M had 38 males and 22 females in aged 25-63 years with an average of 54.03±7.81 years, and 40 males and 20 females in Group-H, aged 21-60 years, with an average of 51.78±6.85 years. No significant difference was observed in the general data of the four groups of patients, which was comparable (Table-I).

**Table-I:** Comparative analysis of the general data of the two groups (*χ̅*±*S*) n=60.

Indicators	Group-R	Group-L	Group-M	Group-H	F/χ^2^	p
Gender (Male, %)	41	43	38	40	0.988	0.804
Age (years old)	53.83±7.02	52.75±7.35	54.03±7.37	51.78±6.72	1.288	0.279
Course of disease (years)	4.77±1.01	4.52±1.08	4.70±1.01	4.68±1.03	0.631	0.595
BMI (kg/m^2^)	33.25±2.54	33.65±2.07	32.85±2.18	33.18±2.29	1.245	0.294
Hypertension (cases, %)	25	23	21	23	0.564	0.905
Hyperlipidemia (cases, %)	23	16	21	18	2.203	0.531
Smoking (cases, %)	32	34	30	28	1.335	0.721
Drinking (cases, %)	26	23	27	22	1.173	0.760

p>0.05.

### Ethical Approval

The study was approved by the Institutional Ethics Committee of Baoding NO.1 Central Hospital(No.:[2021]021; date: June 10, 2021), and written informed consent was obtained from all participants.

### Inclusion criteria:

- Patients who meet the diagnostic criteria for rheumatoid arthritis (RA) formulated by the American College of Rheumatology (ACR).^8^
- Patients aged 18-65 years.
- Patients with complete clinical data.
- Patients who agreed to be included in the study and signed informed consent.

### Exclusion criteria:

- Patients with severe underlying diseases such as heart, liver, kidney and other organ dysfunction
- Patients who are mentally abnormal and unable to cooperate with the completion of the study.
- Patients with diseases affecting the study such as malignant tumors, infectious diseases, chronic inflammatory diseases, and autoimmune diseases.
- Patients with systemic failure.
- Patients who are unable to cooperate with the study satisfactorily.

### MSUS method

Philips full digital color Doppler ultrasound diagnostic instrument with linear array probe was used, and the ultra-wideband band was selected as L12-550 mm. During the examination, the diseased joint was kept in a relaxed state and placed in a functional position. The transverse and longitudinal scanning of the diseased joint was carried out with an ultrasonic probe, and the diseased joint was observed from different angles. First, gray-scale ultrasound was used to observe the synovial sonographic situation, and the thickness of the synovial membrane, cartilage and the depth of intra-articular effusion were measured. Each section was checked three times and the average value was taken. The examination results were judged by two experienced sonographers. In case of disagreement on the diagnosis, another physician will verify it.

### Laboratory examination methods

Five mililiter of fasting blood was collected from the subjects in the morning and centrifuged at 3000 r/minutes to extract the upper plasma. An automatic biochemical analyzer was utilized to detect bone metabolism indicators of patients with RA; A dynamic erythrocyte sedimentation meter was used to detect erythrocyte sedimentation rate, while immune enhancement turbidimetry was used to detect the level of rheumatoid factor in patients.

### Observation indicators:

- MSUS semi-quantitative grading: four indicators were evaluated according to the MSUS semi-quantitative scoring system^9^, including:
- *Bone erosion:* zero point for no bone erosion, one point for rough surface of bone cortex but no defects, two points for obvious bone defects and three points for large bone defects;

### Synovial hyperplasia

zero point for normal conditions, one point for synovial hyperplasia but limited to the angle of the articular surface but not exceeding the line connecting the highest point of the bone surface, two points for periosteal hyperplasia exceeding the line connecting the highest point of the bone surface but not extending to the backbone, three points for hyperplasia extending to the backbone;

### Joint effusion

zero point for no joint effusion, one point for a small amount of effusion, two points for medium amount of effusion, and three points for large amount of effusion;

### Intrasynovial blood flow signal

zero point for normal no blood flow, one point for single blood flow signal, two points for blood flow signal less than 50% of the synovial area, three points for blood flow signal greater than or equal to 50% of the synovial area); Bone metabolism indicators: Bone salt metabolism indicators of patients in the four groups were compared and analyzed, including procollagen Type-1 N-terminal propeptide (P1NP), bone-specific alkaline phosphatase (BALP) and osteoprotegerin (OPG) and tartrate-resistant acid phosphatase (TRAC);

Comparative analysis of the differences in the levels of rheumatoid factor (RF) and erythrocyte sedimentation rate (ESR) among the four groups; 4) Correlation between MSUS semi-quantitative grading and bone metabolism indicators, RF and ESR.The maximum follow-up time for patients in both groups was 6 months. And case data collection ceased in December 2022.

### Statistical analysis

All data were analyzed with SPSS 20.0 software, and measurement data were expressed as (*χ̅*±*S*). Analysis of variance was employed for inter-group data analysis, and χ^2^ test was used for rate comparison. The correlation between MSUS semi-quantitative grading and bone salt level, rheumatoid factor and erythrocyte sedimentation rate were expressed by the Pearson correlation coefficient. P<0.05 indicates a statistically significant difference.

## RESULTS

The MSUS semi-quantitative grading scores of the four groups are shown in Table-II, indicating that the scores of bone erosion, synovial hyperplasia, joint effusion and intrasynovial blood flow signal in Group-H were significantly higher than those in Group-R, Group-L, Group-M, with statistically significant differences (p=0.00).

**Table-II:** Comparative analysis of MSUS semi-quantitative grading indicators among the four groups (*χ̅*±*S*) n=60.

Group	Bone erosion	Synovial hyperplasia	Joint effusion	Intrasynovial blood flow signal
Group-R	0.73±0.71	0.85±0.66	0.57±0.50	0.77±0.70
Group-L	1.42±0.83	1.25±0.60	1.03±0.58	1.40±0.81
Group-M	2.12±0.52	1.77±0.46	1.58±0.50	2.15±0.52
Group-H	2.58±0.53	2.28±0.45	2.03±0.49	2.63±0.49
F	90.111	76.508	91.652	99.025
p	0.000	0.000	0.000	0.000

p<0.05.

The comparative analysis of bone metabolism indicators among the four groups showed that the procollagen Type-1 N-terminal propeptide (P1NP), bone-specific alkaline phosphatase (BALP) and osteoprotegerin (OPG) in Group-H were significantly lower than those in Group-R, Group-L and Group-M, with statistically significant differences (p=0.00); The tartrate-resistant acid phosphatase (TRAC) in Group-H was significantly higher than that in Group-R, Group-L and Group-M, with a statistically significant difference (p=0.00) (Table-III).

**Table-III:** Comparative analysis of bone metabolism indicators among the four groups (*χ̅*±*S*) n=60.

Group	P1NP (ng/ml)	BALP (U/L)	OPG (ng/ml)	TRAC (ng/ml)
Group-R	53.17±3.15	17.03±1.34	5.01±0.31	2.30±0.27
Group-L	45.73±3.35	14.52±1.58	4.47±0.29	3.27±0.31
Group-M	40.35±4.43	10.78±1.54	4.02±0.34	3.96±0.31
Group-H	36.55±3.21	8.75±1.57	3.15±0.30	4.87±0.41
F	244.177	362.398	374.751	647.888
p	0.000	0.000	0.000	0.000

p<0.05.

The comparative analysis of RF and ESR levels among the four groups showed that the levels of RF and ESR in Group-H were significantly higher than those in Group-R, Group-L and Group-M, with statistically significant differences (p=0.00).

The correlation analysis showed that the MSUS semi-quantitative score was positively correlated with the levels of P1NP, BALP, OPG, ESR, rheumatoid factor and tartrate-resistant acid phosphatase (p<0.05) (Table-V).

**Table-IV:** Comparative analysis of RF and ESR levels among the four groups (*χ̅*±*S*) n=60.

Group	RF (KU/L)	ESR (mm/h)
R	87.55±4.82	37.73±6.18
L	108.54±9.94	46.75±6.06
M	123.60±6.89	65.07±7.76
H	136.85±7.12	75.88±7.45
F	488.261	376.139
p	0.000	0.000

p<0.05.

**Table-V:** Correlation analysis between MSUS semi-quantitative grading and bone metabolism indicators, RF and ESR (*χ̅*±*S*) n=60.

Indicators	r	p
P1NP (ng/ml)	0.765	0.000
BALP (U/L)	0.845	0.000
OPG (ng/ml	0.847	0.000
TRAC (ng/ml)	0.858	0.000
RF (KU/L)	0.865	0.000
ESR (mm/h)	0.814	0.000

## DISCUSSION

According to this study, the musculoskeletal ultrasound semi-quantitative score of patients with RA in Group-H was significantly higher than that in Group-R, Group-L and Group-M, with a statistically significant difference (p<0.05). The results showed that with the progression of the disease, the musculoskeletal ultrasound semi-quantitative score of the patients showed a significantly increased development trend, which was basically consistent with the results of Lautwein et al.^10^ It was also suggested in our study that the procollagen Type-1 N-terminal propeptide (P1NP), bone-specific alkaline phosphatase (BALP) and osteoprotegerin (OPG) in Group-H were significantly lower than those in Group-R, Group-L and Group-M, with statistically significant differences (p=0.00). Tartrate-resistant acid phosphatase (TRAC) in Group-H was significantly higher than that in Group-R, Group-L and Group-M, with a statistically significant difference (p=0.00).

Rheumatoid arthritis (RA) is an autoimmune inflammatory disease mainly manifested by synovial lesions of joints.^11^ In the early stage, it is mainly characterized by pannus formation and synovitis, but in the middle and late stages, it may lead to osteoporosis, cartilage and bone destruction, joint dysfunction and deformity, which seriously affects the quality of life of patients.^12^ Timely and effective early diagnosis and treatment of RA may reverse the changes of synovitis, thereby relieving and controlling the disease. However, RA is complex in terms of pathogenesis. In view of the lack of specific clinical manifestations in the early stage of RA, its early diagnosis and treatment are impractical. To this end, in many clinical discussions in recent years, priority has been given to finding methods for early diagnosis and assessment of RA.^13^ It was believed by Amaral et al.^14^ that the pathological characteristics of RA are mainly synovial exudation and hyperplasia, and excessive formation of synovial vessels could destroy the bone. Therefore, whether patients have synovial hyperplasia, excessive vascular deformation and bone erosion should be determined as soon as possible, which is conducive to the diagnosis of RA.

With the continuous expansion of the clinical application of ultrasound technology, color Doppler ultrasound boasts the advantages of a high diagnostic rate and no wound safety in the examination of RA.^15^ In contrast, musculoskeletal ultrasound has the characteristics of high frequency, clear imaging, simple operation, no trauma or radiation damage^16^, mainly including several parameters such as two-dimensional gray-scale ultrasound, three-dimensional ultrasound and power Doppler ultrasound. Among them, two-dimensional gray-scale ultrasound can clearly show the joint morphological structure and the surrounding soft tissue structures, which is helpful for real-time monitoring of the changes of the disease, and timely detection of tissue lesions such as bone erosion, joint effusion, and cartilage destruction.^17^ Power Doppler ultrasound can evaluate the degree of joint synovial inflammation based on the changes in specific energy, blood flow and other parameters, thereby improving the accuracy of joint inflammation.^18^

A prospective study by Lautwein et al.^10^ showed suspicious findings in 192 of 560 selected volunteers with abnormal MSUS, of which 43 were diagnosed with RA, and 354 of 560 volunteers had suspicious findings, of which 76 were diagnosed with RA. The detection rate increased with the increase of musculoskeletal semi-quantitative score, and a total score of ≥5 suggested the presence of RA.

The correlation analysis showed that the MSUS semi-quantitative score was positively correlated with the levels of P1NP, BALP, OPG, ESR, rheumatoid factor and tartrate-resistant acid phosphatase (p<0.05). The above results suggest that the pathogenesis of patients with RA has a close bearing on abnormal bone metabolism^19^, and there is also a close correlation between the balance of bone formation and absorption and musculoskeletal ultrasound semi-quantitative grading. It was also suggested in our study that the levels of RF and ESR in Group-H were significantly higher than those in groups R, L and Group-M. Erythrocyte sedimentation rate (ESR) and rheumatoid factor level were positively correlated with musculoskeletal ultrasound semi-quantitative score (p<0.05). RF and ESR are correlated with the activity degree of arthritis, which indirectly indicates that the musculoskeletal ultrasound semi-quantitative score has a certain evaluation value for the activity degree of RA.^20^

### Limitations

It includes a small sample size with limited follow up. . In addition, no comparative study was made with other imaging modalities like MRI, CT and X-ray and musculoskeletal ultrasound. In future we plan to have more patients with prolonged follow up. In addition we also plan to have a comparative study with other imaging modalities to find out the advantages and disadvantages of this examination method.

## CONCLUSION

Musculoskeletal ultrasound can accurately diagnose disease activity in patients with rheumatoid arthritis, and the musculoskeletal ultrasound semi-quantitative grading is closely correlated with bone metabolism indicators, erythrocyte sedimentation rate and rheumatoid factor. It can be combined with laboratory examination to objectively judge the severity of the course of rheumatoid arthritis.

### Authors’ Contributions:

**PS, NW** designed this study, prepared this manuscript, are responsible and accountable for the accuracy and integrity of the work.

**CH** collected and analyzed clinical data.

**XW** Data analysis, significantly revised this manuscript.
